# Protocol for marker-free genome editing in *Saccharomyces cerevisiae* using universal donor templates and multiplexed CRISPR-Cas9

**DOI:** 10.1016/j.xpro.2025.104280

**Published:** 2025-12-22

**Authors:** Darshi Hemani, James H. Grissom, Richard J. Chi

**Affiliations:** 1 Department of Biological Sciences, University of North Carolina at Charlotte, Charlotte, NC 28223, USA

**Keywords:** Cell Biology, Model Organisms, CRISPR

## Abstract

Here, we present a protocol for marker-free genome editing in *Saccharomyces cerevisiae* by combining PCR-based selectable marker cassettes with CRISPR-Cas9. We describe steps for generating gene deletions using MX6 markers and excising the markers by introducing a reusable guide RNA (gRNA)-Cas9 plasmid and universal repair templates, allowing multiplex removal in a single step. Final verification by PCR yields marker-free strains that can be iteratively edited using the same selectable markers.

For complete details on the use and execution of this protocol, please refer to Grissom et al.^1^

## Highlights

• Instructions for marker-free yeast engineering using MX6 PCR cassettes and CRISPR-Cas9
• Steps for excising multiple MX6 markers in a single CRISPR-Cas9 transformation
• Workflow for using reusable gRNA-Cas9 plasmids and universal repair templates in yeast
• Method for iteratively reusing selectable markers after CRISPR-Cas9 marker removal

## Abstract (editor highlights)

Publisher’s note: Undertaking any experimental protocol requires adherence to local institutional guidelines for laboratory safety and ethics.

## Graphical abstract

**Figure:**
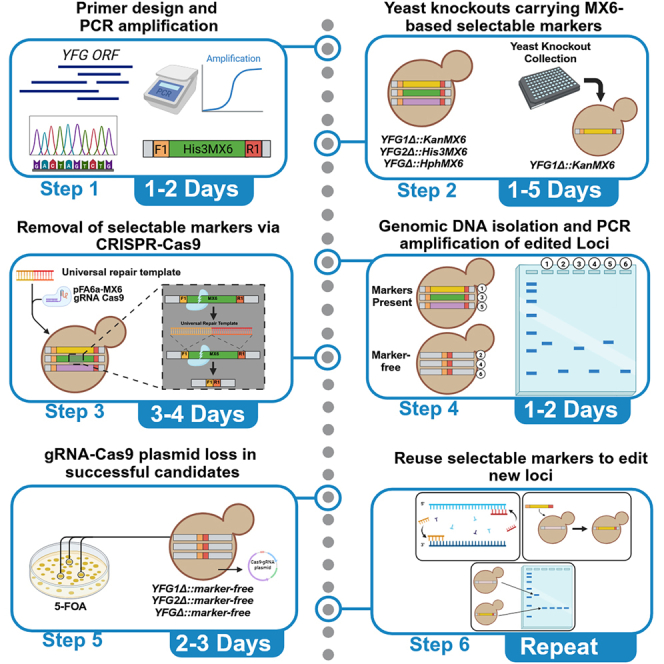

## Before you begin

PCR-based nutritional and drug resistance selectable markers have long been widely used for gene editing in *Saccharomyces cerevisiae* due to their simplicity, versatility, and high efficiency.^2^^,^^3^^,^^4^^,^^5^ However, this approach faces two major limitations: the finite number of available markers and the risk of unintended recombination or marker misintegration in previously edited strains, resulting from shared amplification sequences among PCR-based cassettes. These constraints hinder iterative genome editing and complex strain construction.

CRISPR–Cas9 technology offers a powerful alternative by enabling marker-free genome modification and precise DNA integration in yeast.^6^^,^^7^^,^^8^^,^^9^^,^^10^ Nonetheless, the need to design and validate new guide RNAs (gRNAs) and donor templates for each target locus significantly increases experimental time and cost. Here, we present a hybrid gene editing protocol that combines the strengths of traditional PCR-based methods with the precision of CRISPR–Cas9. The workflow proceeds in two stages. First, yeast strains carrying one or more gene edits are generated using PCR-based MX6 selectable marker cassettes. Next, a universal CRISPR–Cas9 system—consisting of plasmid pJG02 expressing a marker-specific gRNA and a universal donor repair template—is used to excise all MX6based markers, yielding fully marker-free strains.

The pJG02 gRNA was designed to target the conserved *Ashbya gossypii* promoter sequences common to all MX6-derived cassettes, while the donor template is constructed from conserved primer specific flanking regions.^1^^,^^2^^,^^3^ This configuration enables multiplexed removal of all MX6-based markers in a single transformation. Once removed, the markers become immediately reusable for future genome edits.^1^ This system is also compatible with strains from the yeast deletion collection^11^ allowing rapid conversion of existing mutant strains into marker-free versions. Overall, this protocol supports both multiplexed and sequential marker excision, providing an efficient and economical addition to the *Saccharomyces cerevisiae* genome engineering toolkit.

### Innovation

Traditional PCR-based gene disruption in *Saccharomyces cerevisiae* is robust and inexpensive but limited by the finite number of selectable markers and by marker-swapping events when multiple edits are made in the same strain. Existing CRISPR–Cas9 protocols overcome the need for integrated markers but generally require the design and validation of a new gRNA and repair template for each genomic edit, increasing time and cost.

This protocol integrates both strategies into a unified, reusable workflow. First, PCR-based MX6 selectable marker cassettes are used to generate one or more gene deletions using standard F1/R1 primer architecture. Then, a single plasmid expressing Cas9 and a marker-specific gRNA is combined with a universal repair template to remove all MX6 markers present in the genome, either simultaneously or sequentially, thereby returning the strain to a marker-free state.

The key innovations are: (1) reuse of a single gRNA–Cas9 plasmid and universal donor template for multiple rounds of editing, (2) multiplexed excision of several MX6-based markers in a single transformation, and (3) compatibility with existing resources, including the yeast deletion collection and Longtine-based knockouts. Together, these features substantially reduce design effort, streamline construction of complex multi-gene mutants, and expand the flexibility of selectable-marker use in yeast genome engineering.

### Experimental design considerations

1. The protocol is optimized for *S. cerevisiae* strains BY4741 and BY4742, derivatives of the S288C background.
2. It is also compatible with other strains carrying MX6-based selectable markers, including those from the yeast deletion (knockout) collection and pre-existing knockouts generated using the Longtine et al. (1998) method.^2^
  a. In these cases, ensure that you use the correct universal repair template (e.g., U2-D2) corresponding to the selectable marker present in the strain.
  b. Confirm the genotype of the strain and verify the presence and type of selectable markers before proceeding.
  c. Ensure that the gRNA–Cas9 plasmid (pJG02) targets the selectable-marker sequences present in your strain.
3. If using a commercially available knockout strain or a Longtine-based knockout strain, you may skip Steps 1–7 (primer design, PCR amplification, and transformation of selectable marker cassettes) and proceed directly to Step 8.
4. Verify that all plasmids and primers are available and validated before starting the protocol.

### Pre-experimental preparation

5. Media: Prepare YPD, SC–URA, and 5-FOA plates at least 2 days before use to allow adequate drying.
6. Transformation reagents: Prepare fresh LiOAc–TE and 40% PEG3350 if older than 3 months, as degradation reduces transformation efficiency.
7. Safety: Handle chloroform and 5-fluoroorotic acid (5-FOA) in a certified fume hood while wearing gloves, lab coat, and safety goggles; dispose of waste per institutional policy.
8. Timing: Allocate 1–2 days for culture growth and reagent preparation before starting Step 1.

## Key resources table

**Table:** 

REAGENT or RESOURCE	SOURCE	IDENTIFIER
**Chemicals, peptides, and recombinant proteins**		

Agar powder	Thermo Fisher Scientific	9002-18-0
Yeast Nitrogen Base w/o AA, Carbohydrate & w/AS	USBiological	Y2025
CSM powder	Sunrise Science	1300-030
CSM-URA powder	Sunrise Science	1004-100
D-Glucose Anhydrous (Dextrose)	VWR	0188-5KG
Bacteriological Peptone, Ultrapure	Thermo Fisher Scientific	J20048-P5
Yeast Extract (Yeastolate)	USBiological	Y2010
5-FOA	USBiological	F5050
1 M Tris pH 8.0	AmericanBio	AB14043-01000
0.5 M EDTA pH 8.0	AmericanBio	AB00502-01000
Lithium Acetate Dihydrate	Sigma-Aldrich	L6883-1KG
PEG-3350	VWR	0955-1KG
Phusion High Fidelity PCR Kit	Thermo Fisher Scientific	F553L
100 mM dATP	New England Biolabs	N0440S
100 mM dCTP	New England Biolabs	N0441S
100 mM dGTP	New England Biolabs	N0442S
100 mM dTTP	New England Biolabs	N0443S
20% SDS Solution	AmericanBio	AB01922-01000
Ammonium Acetate	AmericanBio	AB00109-01000
DNA (Deoxyribonucleic acid) (from salmon ss)	VWR	MB-103-0025

**Experimental models: Organisms/strains**		

*S. cerevisiae*: Strain background: BY4741, BY4742	Brachmann et al.^12^	N/A
*S. cerevisiae:* BY4742 Knockout Collection	Horizon Discovery	N/A

**Oligonucleotides**		

F1-R1 Repair Template: CGGATCCCCGGGTTAATTAAGGCGCGCCAGATCTGTTTA GGATACTAACGCCGCCATCCAGTTTAAACGAGCTCGAATT C	Grissom et al.^1^	N/A
U2-D2 Repair Template: CGTACGCTGCAGGTCGACGGATCCCCGGGTTAATTAAGG CGCCGCCATCCAGTGTCGAAAACGAGCTCGAATTCATCG AT	Grissom et al.^1^	N/A

**Recombinant DNA**		

Plasmid: pJG02: Cas9 with MX6 gRNA, URA3 (*s.* *cerevisiae*), KanR (*E. coli*)	Grissom et al.^1^	N/A

**Other**		

T100 Thermal Cycler (PCR)	Bio-Rad	1861096
CL 2vCentrifuge	Thermo Fisher Scientific	N/A
5424 R Microcentrifuge	Eppendorf	5424 R
PowerPac HC (electrophoresis)	Biorad	1645052
Digital Dry Bath	Thermo Fisher Scientific	88870001
Incubator GR Con 685GF (Stationary)	VWR	89511-422
Incubator Innova44 (Shaker)	New Brunswick	N/A

## Materials and equipment

**Table:** YPD Plates (per liter)

Reagent	Final concentration	Amount
Agar	25 g/L	25 g
Peptone	20 g/L	20 g
Yeast Extract	10 g/L	10 g
Ultrapure H_2_O		950 mL
40% Dextrose	2%	50 mL
**Total**		**1 L**

***Note:*** Mix thoroughly with a stir bar at 20°C–25°C, then autoclave for 30–45 min. After autoclaving, place the container on a mixer with the temperature set to approximately 45°C. Once the medium has cooled to ∼45°C–50°C, add 50 mL of 40% (w/v) dextrose and mix well with the stir bar. Pour the plates and allow them to dry at 20°C–25°C for ∼2 days. Plates can be stored at 4°C for up to 3 months.

**Table:** SC-URA Plates (per liter)

Reagent	Final concentration	Amount
Yeast Nitrogen Base w/o AA	6.7 g/L	6.7 g
CSM-URA	0.7 g/L	0.7 g
Agar	25 g/L	25 g
Ultrapure H_2_O	N/A	950 mL
40% Dextrose	2%	50 mL
**Total**	**N/A**	**1 L**

***Note:*** Preparation and post-autoclaving steps are identical to those described for YPD plates.

**Table:** 5-FOA Plates

Reagent	Final concentration	Amount
Yeast Nitrogen Base w/o AA	6.7 g/L	6.7 g
CSM	0.7 g/L	0.7 g
Agar	25 g/L	25 g
Ultrapure H_2_O		950 mL
5-FOA	1 g/L	1 g
40% Dextrose	2%	50 mL
**Total**		**1 L**

***Note:*** Preparation and post-autoclaving steps are identical to those described for YPD plates.

**Table:** YPD Liquid (per liter)

Reagent	Final concentration	Amount
Peptone	20 g/L	20 g
Yeast Extract	10 g/L	10 g
Ultrapure H_2_O		950 mL
40% Dextrose	2%	50 mL
**Total**		**950 mL**

***Note:*** Mix thoroughly with a stir bar and aliquot into 250 mL bottles (190 mL per bottle). Autoclave for 30–45 min. Bottles can be stored at 20°C–25°C until use. Before use, add 10 mL of 40% (w/v) dextrose and mix well.

**Table:** TE Buffer (per liter)

Reagent	Final concentration	Amount
1 M Tris pH 8.0	10 mM	10 mL
0.5 M EDTA pH 8.0	2 mM	4 mL
Ultrapure H_2_O		988 mL
**Total**		**1 L**

***Note:*** Mix thoroughly with a stir bar and aliquot into 125 mL bottles (50 mL per bottle). Autoclave for 30–45 min. Bottles can be stored at 20°C–25°C for up to 3 years.

**Table:** LiAC-TE Buffer (per liter)

Reagent	Final concentration	Amount
Lithium Acetate Dihydrate	10 g/L	10 g
1 M Tris pH 8.0	5 mM	5 mL
0.5 M EDTA pH 8.0	0.1 mM	0.2 mL
Ultrapure H_2_O		988 mL
**Total**		**1 L**

***Note:*** Stir to dissolve, then aliquot into 250 mL bottles (200 mL per bottle). Autoclave for 30–45 min. Bottles can be stored at 20°C–25°C for up to 6 months.

**Table:** 40% PEG in LiAC-TE Buffer (per liter)

Reagent	Final concentration	Amount
Lithium Acetate Dihydrate	10 g/L	10 g
1 M Tris pH 8.0	5 mM	5 mL
0.5 M EDTA pH 8.0	0.1 mM	0.2 mL
PEG 3350	200 g/L	200 g
Ultrapure H_2_O		988 mL
**Total**		**1 L**

***Note:*** Using a heat block mixer set to 95°C, slowly add PEG 3350 while stirring until fully dissolved. The solution may remain cloudy for an extended period. Once dissolved, filter-sterilize and aliquot into sterile bottles (100 mL per bottle). Bottles can be stored at 20°C–25°C for up to 3 months**.**

**CRITICAL:** Remake this solution after 3 months; using old PEG 3350 solution significantly reduces yeast transformation efficiency.

**Table:** 10 mM dNTP Stocks

Reagent	Final concentration	Amount
100 mM dATP	10 mM	50 μL
100 mM dCTP	10 mM	50 μL
100 mM dGTP	10 mM	50 μL
100 mM dTTP	10 mM	50 μL
Ultrapure H_2_O		300 μL
**Total**		**500 μL**

***Note:*** Combine reagents in a 1.5 mL microcentrifuge tube and mix thoroughly by pipetting. Aliquot 50 μL into each of ten 1.5 mL tubes and store at −20°C indefinitely. To prepare a 2 mM working stock for PCR, add 200 μL of ultrapure H_2_O to 50 μL of the 10 mM stock. The 2 mM stock can be stored at −20°C indefinitely.

**Table:** gDNA Lysis Buffer

Reagent	Final concentration	Amount
1 M Tris pH 8.0	100 mM	30 mL
0.5 M EDTA pH 8.0	50 mM	30 mL
20% SDS	1%	15 mL
Ultrapure H_2_O		225 mL
**Total**		**300 mL**

***Note:*** Mix thoroughly, then aliquot into 50 mL tubes (50 mL per tube). Tubes can be stored at 20°C–25°C for up to 1 year*.*

**Table:** 7.5 M Ammonium Acetate

Reagent	Final concentration	Amount
Ammonium Acetate	173.43 g/L	173.43 g
0.5 M EDTA pH 8.0	50 mM	300 mL
**Total**		**300 mL**

***Note:*** Adjust the pH to 7.0. To compensate for pH changes, first dissolve ammonium acetate in 100 mL of H_2_O, then bring the final volume to 300 mL after pH adjustment. Mix thoroughly, then aliquot into 50 mL tubes (50 mL per tube). Tubes can be stored at 20°C–25°C for up to 1 year*.*

## Step-by-step method details

### Primer design for the generation of PCR-based selectable markers

**Timing: 30 min**

This section describes how to design primers for generating PCR-based selectable marker cassettes in *Saccharomyces cerevisiae* following the method of Longtine et al.^2^ The primers contain homology regions flanking the gene of interest, enabling targeted replacement through homologous recombination.

Step-by-Step Method.

1. Retrieve the DNA sequence of the target gene.
  a. Navigate to the *Saccharomyces Genome Database* (SGD).
  b. Select the **Sequence** tab and click **Gene/Sequence Resources** as shown in Figure 1A.

**Figure 1:**
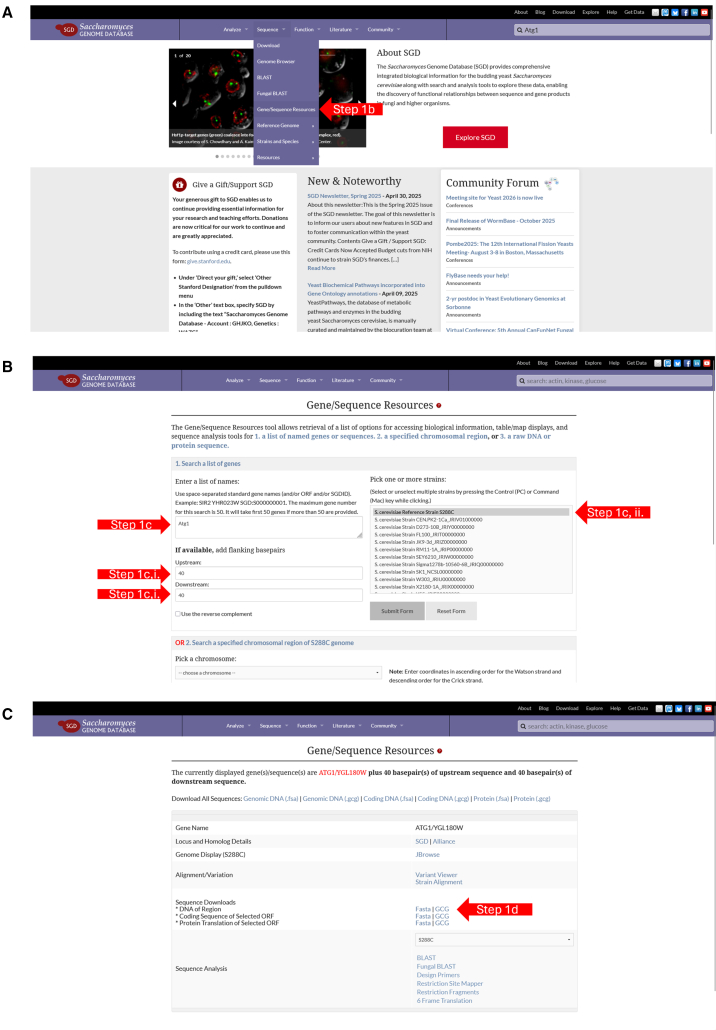
Workflow for retrieving *Saccharomyces cerevisiae* gene and flanking sequences from the Saccharomyces Genome Database (SGD) (A) From the SGD homepage, navigate to the **Sequence → Gene/Sequence Resources** menu (Step 1b). (B) On the Gene/Sequence Resources page, select **Search a list of genes**, enter the desired gene name, and specify the number of upstream and downstream base pairs to retrieve (Steps 1c.i–1c.iii). Choose the appropriate strain(s) from the strain-selection panel (Step 1c.ii), then submit the form. (C) The results page displays gene details and provides sequence download links. Select the **FASTA** format for the desired sequence type (genomic DNA, coding DNA, or protein) to download the flanking-region sequence (Step 1d).
  c. Search for the gene of interest (e.g., ATG1) under “**Enter a list of names”** as shown in Figure 1B.
    i. Enter **40** in both the “**Upstream**” and “**Downstream**” fields in the “if available, add flanking basepairs prompt” and click **Submit Form** as shown in Figure 1B.
    ii. Select Strain “ **S. cerevisiae Reference Strain S288C**” under the “**Pick one or more strain**” form as shown in Figure 1B.
      ***Note:*** Commonly used laboratory yeast strains, including BY4741 and BY4742, are derived from the S288C background.
      **CRITICAL:** For gene deletion, ensure the retrieved DNA sequence includes at least **40 bp upstream and downstream** of the target ORF.
  d. On the resulting page, click the **FASTA** link next to “**∗DNA of Region**” to download the file as shown in Figure 1C.
2. Design primers following Longtine *et al.*^2^
  a. Open the DNA sequence file in **SnapGene** or a similar DNA sequence visualization tool.
  b. Design the **forward (5′) primer** to contain the **F1 sequence** followed by 40 bp immediately upstream of the target gene’s **start codon.**
    ***Note:****F1 sequence*—*5′-****CGGATCCCCGGGTTAATTAA****-3′. e.g., For Atg1 the final primer to order would be 5′-****CGGATCCCCGGGTTAATTAA***TTCAAATCTCTTTTACAACACCAGACGAGAAATTAAGAAA*-3′*.
  c. Design the **reverse (3′) primer** to contain the **R1 sequence** followed by the reverse complement of 40 bp immediately downstream of the gene’s **stop codon.**
    ***Note:****R1 sequence*—5′-**GAATTCGAGCTCGTTTAAAC**-3′. *e.g., For Atg1 the final primer to order would be 5′-***GAATTCGAGCTCGTTTAAAC***GGTCATTTGTACTTAATAAGAAAACCATATTATGCATCAC-3′*.
    **CRITICAL:** Primers must be designed as “F1–upstream homology” and “R1–downstream homology” to ensure correct cassette assembly and integration.
    ***Note:*** Users can adapt this strategy to any gene by substituting the gene-specific 40 bp homology regions while keeping the F1 and R1 sequences identical.

### PCR amplification of selectable marker cassettes

**Timing: 2–3 h**

This section describes how to amplify selectable-marker cassettes using the primers designed above. The PCR product will be used for yeast transformation to generate strains carrying the desired gene deletions or insertions.

3. **Prepare PCR reaction mix** (per 50 μL reaction)

**Table:** 

Reagent	Amount
ddH_2_O	to 50 μL
5X High-Fidelity PCR buffer	10 μL
dNTP’s	0.2 mM
Forward primer (F1Upstream)	0.4 μM
Reverse primer (R1Downstream)	0.4 μM
pFA6a-DNA Template	20 ng (0.004–0.005 pmol)
DMSO	3 μL
High-fidelity DNA polymerase	1 μL

***Note:*** Use a proofreading polymerase such as Phusion or Q5 for high-fidelity amplification.

4. Run PCR in a thermocycler.

**Table:** 

Steps	Temperature	Time	Cycles
Initial Denaturation	98°C	2 min	1
Denaturation	98°C	30 s	30
Annealing	54°C	30 s	
Extension	72°C	2 min 30 s	
Final extension	72°C	10 min	1
Hold	4°C	Infinite hold

5. Analyze PCR products.
  a. Load 5 μL of each reaction on a 1% agarose gel.
  b. Confirm expected cassette size (typically 1.2–1.5 kb, depending on marker).
  c. Purify PCR product using a PCR cleanup kit or ethanol precipitation.

**CRITICAL:** Residual salts or polymerase will reduce transformation efficiency; ensure complete purification.

### Yeast transformation with PCR-amplified selectable marker cassettes

**Timing: 3–4days**

This section describes how to transform *Saccharomyces cerevisiae* with PCR-amplified selectable marker cassettes to generate targeted gene deletions. Yeast cells are rendered competent using lithium acetate and transformed with a mixture of denatured carrier ssDNA and the PCR cassette. Following PEG-mediated transformation and heat shock, cells are plated on selective media to isolate transformants. Integration of the cassette at the correct locus is confirmed by colony PCR after 3–5 days of incubation.

6. Prepare competent yeast cells for Yeast Transformation.
  a. Wash cells with water.
  b. Resuspend pellet in **5 mL sterile ultrapure H**_**2**_**O.**
  c. Centrifuge at **∼2,000 × *g* for 4 min.**
  d. Discard supernatant.
  e. Wash cells with LiOAc–TE solution.
  f. Resuspend pellet in **5 mL LiOAc–TE (100 mM lithium acetate, 10 mM Tris–HCl pH 8.0, 1 mM EDTA).**
  g. Centrifuge at **∼2,000 × *g* for 4 min.**
  h. Discard supernatant.
  i. Preincubate cells in LiOAc–TE.
  j. Resuspend cells in **1/10th original volume (e.g., 1 mL)** LiOAc–TE. Transfer to a microcentrifuge tube and **incubate at 30°C with shaking for 30 min.**
  k. Concentrate competent cells by Centrifugation at **∼5,000 × *g* for 1 min.**
  l. Resuspend pellet in **1/100th original volume (e.g., 0.1 mL)** LiOAc–TE.
    ***Note:*** Resuspended pellet of competent cells can be stored at 4°C for up to 3 days.
  m. Prepare transformation mixture by adding **5 μL of denatured ssDNA (10 mg/mL)** (previously boiled 5 min at 95°C) to a sterile microcentrifuge tube.
    i. Add **0.5–1 μg PCR-amplified DNA cassette.**
      ***Note:*** Keep total DNA mix ≤20 μL. If necessary, reduce template to 5 μg without loss of efficiency.
    ii. Add **50 μL of competent cells** and mix gently by pipetting.
    iii. Incubate transformation mix at **30°C for 30 min.**
    iv. Add **350 μL of 40% PEG 3350** to each tube. Mix thoroughly by pipetting and incubate **1 h at 30°C**.
    v. Add **40 μL DMSO (1/10 volume)** to each tube and mix.
  n. Heat shock at **42°C for 5 min**, then return to 20°C–25°C for 5 min.
  o. Centrifuge at **∼5,000 × *g* for 1 min** and aspirate the supernatant.
  p. Add 100 μL sterile water, resuspend cell pellet and plate on appropriate selective media plates.
  q. Grow in **30°C incubator** for **3–4** days.
7. Confirm transformants.
  a. Streak several colonies onto fresh selective plates to ensure homogeneity.
  b. Verify correct cassette integration by colony PCR using locus-specific primers.

***Note:*** Typical transformation efficiency ranges from 10 to 100 colonies per plate, depending on cassette size and yeast strain.

### Removal of selectable marker cassettes via CRISPR-Cas9

**Timing: 3–4 days**

This procedure describes how to remove MX6-based selectable marker cassettes from edited *Saccharomyces cerevisiae strains* using a reusable gRNA–Cas9 plasmid and a universal repair template, resulting in a fully marker-free strain. The pFA6a-MX6-specific plasmid pJG02 expresses a gRNA that targets conserved *Ashbya gossypii* promoter sequences present in all MX6-derived selectable markers, allowing for multiplexed removal of all markers in a single transformation. This protocol can also be used to remove individual selectable markers sequentially.

8. Yeast transformation with CRISPR-CAS9 and universal template.
  a. **Prepare culture** by sing a sterile applicator, inoculate a single yeast colony into **10 mL of YPD medium.**
    ***Note:*** If the strain carries a plasmid, use the corresponding selective medium instead of YPD. Strains with integrated markers can be grown in YPD.
  b. Inoculate **10 mL of YPD** with 1:10 and 1:100 dilutions of the starter culture. Incubate at **30°C with shaking (200–250 rpm)** 14–16 h.
    ***Note:*** These dilutions typically yield logarithmic-phase cultures the next morning.
  c. **Measure cell density** by Measuring the **OD**_**600**_ of each culture.
    Cultures in logarithmic phase (OD_600_ = 0.4–1.0) are suitable for transformation.
    ***Note:*** If OD_600_ values are too high, dilute to 0.25 and incubate an additional 3–4 h at 30°C with shaking.
  d. **Harvest cells** by centrifuging **10 mL of culture** at **∼2,000 × *g* for 4 min** at 20°C–25°C.
  e. **Preparation of Competent Yeast Cells as described in Step 6** *“Prepare competent yeast cells for Yeast Transformation”.*
    ***Note:*** As in described in Step 6, “Resuspended pellet of competent cells can be stored at 4°C for up to 3 days.”
  f. **Prepare transformation mixture** by adding **5 μL of denatured ssDNA** (previously boiled 5 min and cooled) to a sterile microcentrifuge tube.
    i. Add **0.03–0.05 pmol (250 ng) of pJG02 gRNA–Cas9 plasmid.**
    ii. Add **10 μg of universal repair template**, mix by pipetting.
      ***Note:*** Keep total DNA mix ≤20 μL. If necessary, reduce template to 5 μg without loss of efficiency.
    iii. Add **50 μL of competent cells** and mix gently by pipetting.
  g. Prepare control transformation by adding in a separate tube, add **5 μL denatured ssDNA.**
    i. Add **0.03–0.05 pmol (250 ng) Cas9 plasmid (no repair template)** and **10 μL sterile ultrapure H**_**2**_**O.**
    ii. Add **50 μL competent cells** and mix by pipetting.
      ***Note:*** The control transformation lacks the repair template; repeated Cas9 cutting is toxic and should result in few or no colonies.
  h. **Incubate transformation mixes** at **30°C for 30 min.**
  i. Add 350 μL of 40% PEG 3350 to each tube. Mix thoroughly by pipetting and incubate 1 h at **30°C**.
  j. **Heat shock** buy adding **40 μL DMSO (1/10 volume)** to each tube and mix.
    i. Heat shock at **42°C for 5 min**, then return to 20°C–25°C for 5 min.
    ii. Centrifuge at **∼5,000 × *g* for 1 min** and aspirate the supernatant.
  k. Resuspend cell pellets in **300 μL sterile ultrapure H**_**2**_**O.**
    i. Spread entire reaction (∼400 μL) onto **synthetic complete medium lacking uracil (SC–URA)** using glass beads.
      ***Note:*** pJG02 carries the URA3 marker, which facilitates later plasmid curing.
    ii. Allow liquid to absorb, then remove beads by pour used beads into 70% ethanol bath.
    iii. Place plates agar side up and incubate at **30°C for 3–4 days.**
      ***Note:*** Transformation efficiency decreases with increasing number of targeted loci. A few colonies on the control plate are expected due to occasional PAM-site mutations.
      **CRITICAL:** Confirm marker removal at each targeted locus by **PCR amplification and gel electrophoresis**. Successful marker excision results in a band shift corresponding to the loss of the *MX6* cassette.

### Preparation of candidate marker-free yeast genomic DNA for PCR

**Timing: 2–3 h**

This procedure describes the isolation of genomic DNA from yeast strains for use in PCR-based verification of marker removal and locus editing.

9. Genomic DNA isolation.
  a. Using a sterile applicator, transfer a single yeast colony from an agar plate to **5 mL YPD medium.**
    ***Note:*** 8–10 colonies are prepared to account for transformation efficiency.
  b. Incubate at **30°C with shaking (200–250 rpm)** 14–16 h to allow cells to reach stationary phase.
  c. **Harvest and wash yeast cells by** centrifuging the culture at **∼2,000 × *g* for 4 min.**
  d. Aspirate the supernatant and resuspend the pellet in **1 mL sterile ultrapure H**_**2**_**O.**
  e. Transfer the suspension to a **1.5 mL microcentrifuge tube.**
  f. Centrifuge at **∼5,000 × *g* for 1 min** and aspirate the supernatant.
  g. Resuspend the pellet in **250 μL lysis buffer.**
    i. Add **acid-washed glass beads** until the liquid level reaches the **500 μL mark.**
      ***Note:*** Zirconia beads may be substituted for glass beads, provided the bead size is 425–600 μm.
    ii. Vortex vigorously at **maximum speed for 15 min** to disrupt cells.
    iii. Add **150 μL 7.5 M ammonium acetate (pH 7.0)** to the tube and mix thoroughly.
    iv. Incubate at **65°C for 15 min.**
  h. Immediately place the tube on **ice for 15 min.**
  i. Add **500 μL chloroform**, and gently invert several times to mix.
    ***Note:*** Exercise caution during mixing; incomplete sealing of the tube can result in chloroform leakage.
  j. Centrifuge at **∼5,000 × *g* for 5 min.**
  k. Carefully transfer the **upper aqueous layer** to a new microcentrifuge tube, avoiding the interphase.
    **CRITICAL:** Avoid aspirating material from the interphase, as this will contaminate the genomic DNA with proteins and debris and future PCR reactions will fail. Repeat steps a-l as needed.
  l. Precipitate genomic DNA by adding 500 μL 100% isopropanol (IPA) to the tube and mix gently.
    i. Centrifuge at **∼5,000 × *g* for 5 min** to pellet the DNA.
    ii. Discard the supernatant, then wash the pellet with **500 μL 70% ethanol (EtOH)** without disturbing it.
    iii. Centrifuge again at **∼5,000 × *g* for 5 min.**
    iv. Discard the supernatant and spin the tube (**5–10 s**) to collect residual liquid.
    v. Using a pipette, carefully remove any remaining EtOH.
    vi. Air-dry the pellet with microfuge tube caps open in a **30°C incubator for 5–10 min.**
    vii. Resuspend the dried DNA pellet in **50 μL TE buffer.**
      ***Note:*** Verify DNA concentration and purity using spectrophotometry or an equivalent method. This protocol typically yields high-quality genomic DNA suitable for PCR. If yield or purity is low, consult the troubleshooting section.

### PCR amplification of the edited loci

**Timing: 2–3 h**

This step verifies successful editing of the gene(s) of interest following yeast transformation. PCR amplification is performed using genomic DNA extracted from the candidate marker-free strains. For each target locus, use a **forward flanking primer** homologous to the region **50–100 bp upstream of the start codon** and the **“R1” primer** originally used to generate the deletion cassette.

Amplification with these primers confirms both **specificity of the cassette** and its **correct integration** at the intended locus. Any additional loci previously modified in the strain should also be tested via PCR to confirm proper integration of selectable markers and the absence of unintended rearrangements.

***Note:*** Alternatively, the inverse primer set can be used: the “F1” primer employed during cassette construction, paired with a reverse flanking primer homologous to a region downstream of the stop codon.

10. PCR amplify edited loci buy Preparing PCR reaction Mix.

**Table:** 

Reagent	Amount
ddH_2_O	63 μL
10X DreamTaq Buffer	20 μL
dNTP’s	0.2 mM
Forward Primer 1	20 μM
Reverse Primer 2	20 μM
DNA Template	20 ng (0.004–0.005 pmol)
DMSO	3 μL
DreamTaq DNA Polymerase	1 μL
  a. Run PCR in a thermocycler.

**Table:** 

Steps	Temperature	Time	Cycles
Initial Denaturation	98°C	2 min	1
Denaturation	98°C	30 s	30
Annealing	54°C	30 s	
Extension	72°C	2 min 30 s	
Final extension	72°C	10 min	1
Hold	4°C	Infinite hold

    ***Note:*** After completing the PCR, analyze products by agarose gel electrophoresis to confirm the expected band size. If no bands are detected or if the bands appear smeared, consult the troubleshooting section.

### gRNA-Cas9 plasmid loss in successful candidates

**Timing:3–4days**

This step results in loss of the CRISPR–Cas9 plasmid following successful removal of selectable markers from the yeast genome. Because the gRNA–Cas9 plasmid carries a **URA3** selectable marker, plasmid loss can be achieved by **counterselection on agar medium containing 5-fluoroorotic acid (5FOA)**, which is toxic to cells expressing URA3.

**CRITICAL:** If additional marker-free edits are planned in the same yeast strain, this step must be completed before introducing new selectable markers. Otherwise, Cas9 will degrade the marker cassettes before they integrate into the genome, causing transformation failure.

11. gRNA-Cas9 Plasmid Loss.
  a. Using a sterile applicator, inoculate a single yeast colony into **10 mL YPD medium.**
  b. Incubate 14–16 h at **30°C with shaking (200–250 rpm).**
  c. The following day, prepare **1:10 and 1:100 dilutions** of the overnight culture in **10 mL fresh YPD**, and incubate until cultures reach the **logarithmic phase** (OD_600_ = 0.4–1.0).
    ***Note:*** A 1:10 and 1:100 dilution is typically sufficient to achieve log-phase growth by the following day.
  d. Prepare **1:10, 1:100, and 1:1000 dilutions** of the log-phase culture in 10 mL YPD.
  e. Label three **5-FOA plates** according to the dilution series.
  f. Transfer **500 μl** cell suspensions directly onto the plates using a pipette.
  g. Add glass beads to each plate and gently move plates side to side on the benchtop to evenly disperse the liquid.
  h. Allow the liquid to absorb fully into the agar.
  i. Remove glass beads by tipping the plate over a container of **70% ethanol (EtOH).**
  j. Place plates agar side up and incubate at **30°C for 3–4 days.**
    ***Note:*** Colonies that grow on 5-FOA plates have successfully **lost the gRNA–Cas9 plasmid**.
    ***Note:*** To confirm plasmid loss, streak resulting colonies onto **synthetic complete medium lacking uracil (SC–URA)**. Only strains that have retained the plasmid will grow on SC–URA plates.

## Expected outcomes

Upon successful completion of this protocol, researchers should obtain marker-free *Saccharomyces cerevisiae* mutants. The procedure is optimized for the simultaneous removal of multiple selectable markers, while remaining flexible for sequential removal of individual markers. This approach can be applied iteratively using the same marker cassettes, provided that the gRNA– Cas9 plasmid is removed from the strain after each round of marker excision.

Our results indicate that the efficiency of single-marker removal is approximately 90%,^1^ whereas the efficiency of removing three markers simultaneously may decrease to ∼50%.^1^ Notably, in strains harboring multiple markers, all candidate colonies were either completely marker-free or retained all markers, suggesting that the gRNA–Cas9 plasmid does not exhibit preferential targeting among *MX6*-based selectable-marker cassettes.

Consistent with standard yeast transformation efficiencies, researchers should expect to observe >100 colonies following both (1) cassette integration via homologous recombination and (2) single-marker removal via CRISPR–Cas9 after the recommended incubation periods. The number of colonies typically decreases as the number of markers targeted increases. If transformation efficiency is markedly low, potential issues may include suboptimal cassette integration, degraded DNA, or reagent failure; refer to the troubleshooting section for guidance.

After introducing the gRNA–Cas9 plasmid and universal repair template into yeast containing selectable markers, the control transformation (containing only the gRNA–Cas9 plasmid) may still produce a small number of colonies. This is expected, as some cells may acquire mutations that disrupt gRNA binding via non-homologous end joining (NHEJ) or PAM-sequence point mutations following Cas9-induced cleavage. Typically, the number of colonies on the control plate should not exceed 10%–20% of the colonies observed on the experimental (transformant) plate, confirming efficient marker removal.

## Limitations

A primary limitation of this protocol involves reduced transformation efficiency during repeated sequential marker-free edits in the same yeast strain. Each genomic edit employs selectablemarker cassettes containing conserved “F1” and “R1” primer sequences, which facilitate reuse of a universal repair template for marker removal via CRISPR–Cas9. However, retention of these sequences after marker excision increases the likelihood of marker-swapping events in strains carrying multiple knockouts. This phenomenon occurs when similar amplification sequences (such as the *F1* and *R1* regions) cause a newly introduced cassette to replace an existing marker rather than integrate at the intended locus.

To minimize this risk, candidate strains containing multiple edits should be verified either by testing growth on single dropout media plates or on dropout media lacking all relevant nutrients and/or containing all corresponding drug markers.

We observed that after three sequential marker-free edits, the efficiency of obtaining correct candidates begins to decline markedly.^1^ PCR analysis of genomic DNA from these strains often reveals marker-swapping as the underlying cause. To mitigate this, we recommend multiplexed marker removal, which—although still subject to occasional swapping—reduces the number of transformations required to generate fully marker-free strains. For example, creating three sequential marker-free edits requires at least six yeast transformations, whereas multiplexed removal of three markers requires only four.

For researchers preferring sequential editing, we suggest extending the length of the PCR cassette to improve locus specificity. After generating a single gene edit in a wild-type strain, design flanking primers 200–300 bp outside the edited locus to amplify a longer cassette for subsequent transformations. This approach enhances targeting precision during future sequential edits and helps prevent unwanted recombination between shared cassette sequences.

## Troubleshooting

### Problem 1

No colonies appear on the transformation plate, or colonies are present on the control plate (related to Steps 7 or 8).

Transformation of DNA into yeast occasionally yields unwanted colonies on control media plates or no colonies at all.

The presence of colonies on the control plate typically indicates bacterial or fungal contamination in one or more transformation components.

A complete absence of colonies on the experimental plate suggests issues with media composition, selectable-marker integrity, or yeast cell viability.

### Potential solution

• Extend incubation time.

Some genomic edits impose physiological stress on yeast cells, resulting in slower growth than wild type. Colonies may require up to 5–7 days to appear.

• Use fresh components.

Fungal or bacterial colonies on control plates usually arise from contamination in transformation reagents. Inspect all components—water, YPD, LiOAc–TE, 40% PEG, and DMSO—for visible growth or sediment. If contamination is not visible, replace the water source first, as it is the most common culprit. If the problem persists, replace all solutions and consumables (e.g., pipette tips) with newly autoclaved materials. Employ proper aseptic technique, such as working near a Bunsen burner, to minimize contamination risk.

• Confirm selectable marker integrity.

Improperly amplified marker cassettes can fail to integrate into the genome. Verify that the PCR product used for transformation displays a single, distinct band on an agarose gel.

Smearing or multiple bands indicate incorrect amplification and require repeating the PCR.

• Check media plate quality and compatibility.

Confirm that plates are appropriate for the transformation performed—for example, when transforming a **His3MX6** cassette, the medium should contain all necessary nutrients except histidine. Old or desiccated plates (>3 months old) significantly reduce transformation efficiency and should be remade. Also inspect stored plates for condensation, which can promote microbial contamination; wipe and dry storage containers as needed.

• Ensure yeast freshness.

Yeast maintained on agar plates at 4°C should be periodically restreaked onto fresh medium. Avoid using strains stored on the same plate for more than 3 months.

### Problem 2

Low yield from genomic DNA isolation (related to step 9).

Yeast genomic DNA isolation typically results in a large yield of DNA (500 ng/μL or higher). Low DNA concentration usually indicates insufficient cell input, degradation or expiration of reagents, or loss of material during extraction and phase separation.

### Potential solution

• Remake buffers and solutions.

If the genomic DNA lysis buffer is near or past its one-year expiration, lysis efficiency may decrease. Old or improperly stored reagents are the most common cause of low yield. Check the pH of the **7.5 M ammonium acetate** regularly; deviations from pH 7.0 can reduce DNA precipitation efficiency.

• Verify cell density before lysis.

Cells should be in **stationary phase** before harvesting. Insufficient biomass results in low total DNA yield. Measure OD_600_ before lysis—values should be **≥ 5.0** to ensure adequate cell density.

• Handle the chloroform extraction carefully.

During phase separation (Step 9), avoid transferring any material from the interphase to the new tube. Contamination with interphase material reduces DNA recovery and purity. It is preferable to leave a small amount of the upper aqueous layer behind rather than risk collecting debris.

### Problem 3

No bands or smeared bands following amplification of genomic DNA by PCR (related to Step 9) Failed PCR amplification can result in no visible bands or smeared bands on an agarose gel.

A lack of bands usually indicates suboptimal template DNA concentration or missing/expired reaction components, whereas smeared bands often result from incorrect annealing temperatures or primer mismatches during amplification.

### Potential solution

• Check the concentration of template DNA.

Using too high a concentration of template DNA can inhibit the reaction, while too little template may not yield enough product for visualization. Dilute genomic DNA to a working concentration of 20–100 ng/μL for optimal amplification.

• Verify annealing temperature and primer compatibility.

To minimize band smearing, set the annealing temperature to 3°C–5°C below the lower of the two primer melting temperatures (T_m_). Ensure that both primers have T_m_ values within 5°C of each other to promote uniform annealing.

• Confirm PCR components and reagent integrity.

No bands may also result from missing or degraded reagents. Verify that all PCR components (e.g., buffer, dNTPs, primers, polymerase, and water) are present and not expired. Always double-check reaction setup before thermocycling.

### Problem 4

High number of colonies on the control plate following gRNA–Cas9 transformation (related to Step 8). Under normal conditions, repeated double-strand breaks (DSBs) generated by Cas9 are toxic to yeast cells lacking a repair template. A small number of colonies on the control plate is expected, as some cells survive through non-homologous end joining (NHEJ) of the cut site or point mutations within the PAM sequence.

However, if the number of colonies on the control plate is comparable to that on the transformation plate, this indicates reduced activity or loss of efficiency of the Cas9–gRNA plasmid.

### Potential solution

• Use a fresh stock of the gRNA–Cas9 plasmid.

Repeated freeze–thaw cycles can cause plasmid degradation, leading to diminished editing efficiency. Prepare aliquots of the plasmid to avoid repeated thawing, or generate a new plasmid stock for future transformations.

• Verify the background yeast strain.

If the strain lacks the selectable marker targeted by the gRNA–Cas9 plasmid, both control and transformation plates will show similar colony numbers, as Cas9 will not create DSBs at the intended locus. Confirm the presence of the marker by patch testing colonies on selective dropout medium. If many colonies are present on the control plate despite a confirmed marker, sequence the marker region to check for point mutations in the gRNA target or PAM site that could prevent Cas9 cutting.

### Problem 5

Incorrect target integration following multiple edits in the same yeast strain (related to Step 7). Because this protocol relies on a universal repair template, the conserved “F1” and “R1” sequences must remain in the genome to allow subsequent marker removal. In yeast strains undergoing multiple genomic edits, these homologous regions can mediate “marker-swapping” events via homologous recombination. In such cases, a newly introduced selectable marker may integrate into the locus of a previous edit, replacing an existing marker rather than integrating at the intended target site. A similar outcome may also occur during repeated rounds of marker-free editing, since the retained *F1/R1* sequences increase the probability of recombination at unintended loci. The likelihood of incorrect marker integration rises with the number of sequential edits performed in the same strain.

### Potential solution

• Increase the number of candidate colonies screened.

If incorrect integrations are frequently observed, expanding the number of screened colonies can often identify at least one correctly edited clone. Even when a selectable marker integrates into multiple loci—including the correct one—the gRNA will still target all instances of the marker during the marker-removal step, ultimately yielding a marker-free strain.

• Extend the length of the selectable marker cassette.

Increasing the size of the gene-specific flanking regions improves locus specificity and reduces the frequency of “marker swapping.” To do this, first generate a knockout of the target gene in wild-type yeast. Then, using primers located 400–500 bp outside the start and stop codons, amplify the knockout region by PCR. This longer PCR product can serve as the selectable-marker cassette for transformation into the previously edited strain. We have found this approach significantly increases correct-targeting efficiency and recommend it if standard editing efficiency begins to decline.

## Resource availability

### Lead contact

Further information and requests for resources and reagents should be directed to and will be fulfilled by the lead contact, Richard J. Chi (richard.chi@charlotte.edu).

### Technical contact

Further technical information can be directed to and will be fulfilled by the technical contact, James H. Grissom (james.grissom@lr.edu).

### Materials availability

Plasmids in this study (pJG02) are planned to be deposited to Addgene. Information regarding the catalog number/unique identifier can be obtained through the lead contact upon their upload.

### Data and code availability

This study did not generate/analyze datasets/code.

## Acknowledgments

We are grateful to the members of the Chi Lab for their thoughtful discussions and comments on this manuscript. We also thank undergraduate researchers Alyssa Lucero, Carrie Rapier, Jordan Swaby, and Briggs Yoder for their direct contributions to developing this project. This work was supported by 10.13039/100000001National Science Foundation (NSF) grant 2028519 to R.J.C. and by the UNC Charlotte Faculty Research Grants Program to R.J.C.

## Author contributions

Study conception and design, R.J.C. and J.H.G.; data collection, J.H.G. and D.H.; analysis and interpretation of results, J.H.G. and D.H.; manuscript preparation, J.H.G., D.H., and R.J.C. All authors reviewed the results, contributed to the discussion, and approved the final version of the manuscript.

## Declaration of interests

The authors declare no competing interests.
